# Hilar mossy cells of the dentate gyrus: a historical perspective

**DOI:** 10.3389/fncir.2012.00106

**Published:** 2013-01-09

**Authors:** Helen E. Scharfman, Catherine E. Myers

**Affiliations:** ^1^New York University Langone Medical CenterNew York, NY, USA; ^2^Center for Dementia Research, The Nathan Kline Institute for Psychiatric ResearchOrangeburg, NY, USA; ^3^Veterans Affairs Medical Center - New Jersey Healthcare SystemEast Orange, NY, USA; ^4^New Jersey Medical School, University of Medicine and Dentistry of New JerseyNewark, NJ, USA

**Keywords:** dentate gyrus, hippocampus, interneuron, granule cell, mossy fibers, excitotoxicity, vulnerability

## Abstract

The circuitry of the dentate gyrus (DG) of the hippocampus is unique compared to other hippocampal subfields because there are two glutamatergic principal cells instead of one: granule cells, which are the vast majority of the cells in the DG, and the so-called “mossy cells.” The distinctive appearance of mossy cells, the extensive divergence of their axons, and their vulnerability to excitotoxicity relative to granule cells has led to a great deal of interest in mossy cells. Nevertheless, there is no consensus about the normal functions of mossy cells and the implications of their vulnerability. There even seems to be some ambiguity about exactly what mossy cells are. Here we review initial studies of mossy cells, characteristics that define them, and suggest a practical definition to allow investigators to distinguish mossy cells from other hilar neurons even if all morphological and physiological information is unavailable due to technical limitations of their experiments. In addition, hypotheses are discussed about the role of mossy cells in the DG network, reasons for their vulnerability and their implications for disease.

## “Moss” and “mossy cells”

Ramon y Cajal was the first to describe the unusual large boutons of granule cell axons from his studies of the rabbit or guinea pig (Ramon y Cajal, 1911). He gave these axons the name “mossy fibers” because the giant terminals of granule cells, occurring periodically along the granule cell axons, gave the axons the appearance that they were covered in moss (Ramon y Cajal, 1911). The adjective “mossy” is also used for other fiber systems (e.g., cerebellar mossy fibers) but in the hippocampus the only cells with mossy fibers are granule cells.

Many decades later, electron microscopy was used to describe mossy fiber boutons in more detail, and showed that they are complex, large terminals, densely packed with synaptic vesicles (Blackstad and Kjaerheim, 1961; Laatsch and Cowan, 1966). These boutons innervate equally complex structures on the proximal apical dendrites of CA3 pyramidal cells, called “complex spines” or “thorny excrescences,” a name that reflects the similarity to thorny excrescence of plants, which are complex protrusions that emerge from the main stem. The remarkable complexity of mossy fiber boutons and thorny excrescences—much more intricate than most pre- and postsynaptic structures—is not unique to area CA3, however. Complex, large mossy fiber boutons of granule cells are also abundant in the hilus, where they contact thorny excrescences on the proximal dendrites and somata of a subset of hilar neurons.

**Figure 1:**
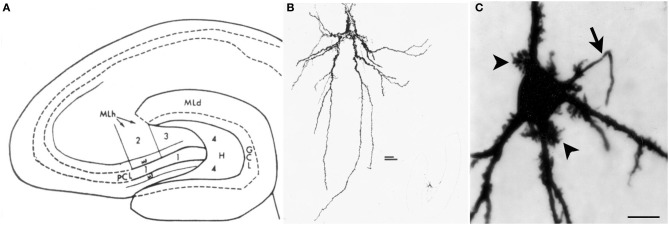
**Introduction to the hilus and mossy cells. (A)** A schematic of the rat hippocampus from Amaral (1978) in horizontal section shows the location of the hilus (zone 4). GCL, granule cell layer; H, hilus; ML, molecular layer; MF, mossy fiber; PCL, pyramidal cell layer. **(B)** A drawing of a mossy cell from the same study (Amaral, 1978). **(C)** A mossy cell that was physiologically-identified in hippocampal slices from an adult male rat and filled with Neurobiotin from Scharfman et al. (2001). An arrow points to the axon; arrowheads point to thorny excrescences. Calibration = 20 μm.

The neurons of the hilus with thorny excrescences were named “mossy cells” because their appearance resembles a cell covered in moss (Amaral, 1978) (Figure 1). Hilar mossy cells with these characteristics have now been described in numerous mammalian species besides rats and mice, including guinea pig (Scharfman and Schwartzkroin, 1988), gerbil (Kotti et al., 1996), hamster (Murakawa and Kosaka, 2001), and primates (Seress and Mrzljak, 1992; Seress and Ribak, 1995).

Before describing mossy cells in more detail, it is important to clarify nomenclature of the hilar region (see also, Amaral et al., 2007; Scharfman and Witter, 2007). Originally the hilus was described by other terms: area H5 (Rose, 1926), CA4 (Lorente De Nó, 1934), or the polymorphic zone, and it was debated if the area between the granule cell layer and area CA3 should be addressed as a single area or multiple subregions (discussed in Amaral, 1978). At the present time, “hilus” has replaced these terms for the most part, although the term “polymorphic zone” is still applicable to the dentate gyrus (DG) because the hilus is a polymorphic layer if one defines the DG as a structure composed of a molecular layer, cell layer, and polymorphic layer.

**Figure 2:**
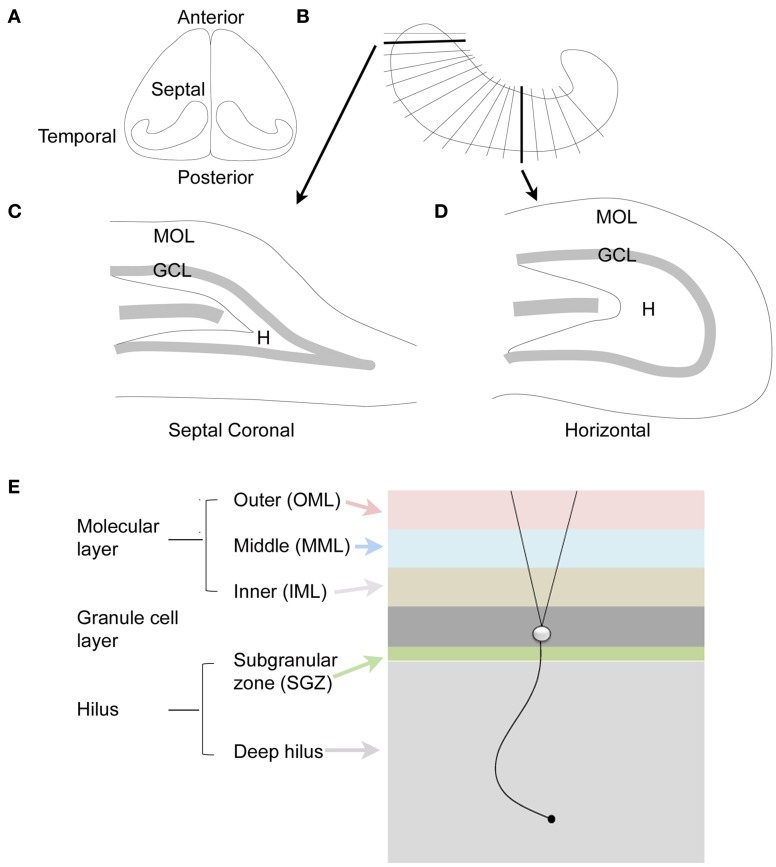
**The dentate gyrus of the rodent. (A)** Dorsal view of the rodent hippocampus. **(B)** A schematic of the lamellar organization of the hippocampus. Two sections are shown in the coronal plane and horizontal plane as indicated by the arrows. **(C)** A coronal section from septal hippocampus is illustrated. MOL, molecular layer; GCL, granule cell layer; H, hilus. **(D)** A horizontal section from temporal hippocampus is illustrated. **(E)** The laminar organization of the DG is illustrated, with a single granule cell to show the orientation of dendrites and the granule cell axon, which is called a mossy fiber. The molecular layer is divided into three zones that are approximately the same width: outer molecular layer (light red); middle molecular layer (light blue); and inner molecular layer (light brown). The granule cell layer (dark gray) has several layers of densely-packed granule cells. Below the granule cell layer is a small subgranular zone (light green) containing hilar neurons and precursors of granule cells. The hilus includes the subgranular zone and a larger area that ends with area CA3c. The zone near CA3c is sometimes called the deep hilus (light gray).

Another aspect of nomenclature that is important relates to the terms for the different parts of the hippocampus: septal vs. temporal poles. In the rodent, the septal pole is located in the dorsal part of the forebrain, and is more anterior than the rest of the hippocampus; the temporal pole is located in the ventral part of the forebrain and is more caudal or posterior (Figure 2). However, septal and dorsal hippocampus are not necessarily synonymous, because some of the hippocampus that is located in the dorsal part of the forebrain is relatively caudal and is not very close to the septum. Therefore, it is preferable to discuss the DG with terms such as septal or temporal rather than dorsal and ventral (Scharfman and Witter, 2007). On the other hand, a section cut in the horizontal plane in the dorsal part of the brain is best discussed as dorsal, since it contains both septal areas and more caudal areas.

Since the landmark paper by Amaral (1978), that described hilar neurons from Golgi-stained tissue of the rat, much more has become known about the basic structural and functional characteristics of mossy cells, and their potential contribution to the DG and CA3 network. Below we discuss the fundamental characteristics of mossy cells, and then discuss the hypotheses about their function, vulnerability, and implications for disease.

## Diverse characteristics of mossy cells and the question they raise: what is a mossy cell?

### Early studies of the axon projection of mossy cells and the questions they raised

**Figure 3:**
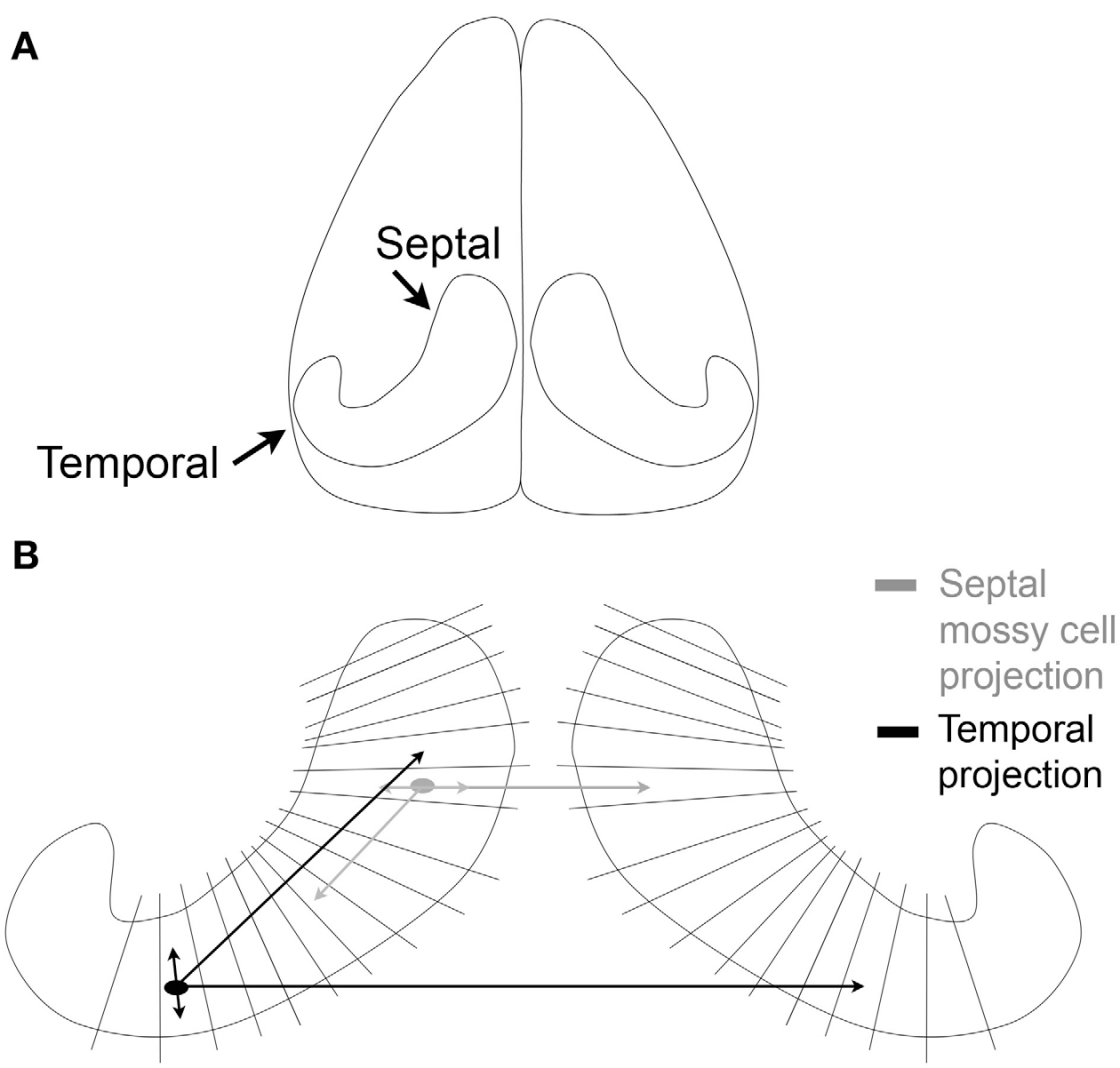
**The commissural/associational (C/A) pathway. (A)** An illustration of the brain viewed from above shows the orientation of the hippocampus of the rodent. **(B)** The lamellae of the hippocampus are illustrated schematically. Mossy cells located near the temporal pole of the hippocampus (black) project to septal areas and to the temporal hippocampus contralaterally. The projections that are local terminate primarily in the hilus; the projections to distant lamellae terminate primarily on granule cell dendrites in the inner molecular layer. Mossy cells located in septal hippocampus (gray) have similar local and distant projections but the distant projection does not extend as far from the cell body as it does for temporal mossy cells.

Before much was known about hilar mossy cells, a great deal of work was already being conducted to understand the commissural projection of large hilar neurons—which later became identified as the axon projection of mossy cells, as well as some other types of DG neurons. The axon projection was called the commissural/associational (C/A) pathway and projected to distal “lamellae” of the DG ipsilaterally and the contralateral DG. It formed the major source of afferents to the inner molecular layer (Figures 2, 3; Ribak et al., 1985). Most of these studies were conducted in rats and were based on tract-tracing techniques. Some of the data showed remarkably specificity: the contralateral projection to the inner molecular layer targeted a similar location along the septotemporal axis as the cell bodies of origin (“homotopic”; Figure 3).

Most of these studies suggested that the cells of origin of the C/A pathway were large neurons in the hilus (large referring to the size of the cell body). However, it was never entirely clear that these large neurons were exclusively mossy cells or that large hilar neurons were only mossy cells. One reason to be cautious was that some rather small-sized hilar neurons appeared to contribute to the C/A projection (Ribak et al., 1985). These relatively small hilar neurons seemed unlikely to be mossy cells based on the idea that mossy cells have a large soma.

Might other neurons besides mossy cells contribute to the C/A pathway? Did some mossy cells in fact have small cell bodies? Additional studies supported both ideas. Regarding the heterogeneity of neurons contributing to the C/A pathway, it became clear that other hilar neurons than mossy cells have a commissural projection. One study was physiological: in the anesthetized rat, electrical stimulation of the commissure could inhibit the granule cell population spike evoked by a prior stimulus to the perforant path (Douglas et al., 1983). These data suggested that there was a GABAergic contribution to the commissural pathway, which would be unlikely to be mossy cells because they are glutamatergic (although at the time this was debated). Anatomical analyses of commissurally projecting neurons of the DG showed that GABAergic neurons which co-localize somatostatin or neuropeptide Y project contralaterally—although they do not innervate the inner molecular layer in the contralateral hemisphere, like mossy cells (Bakst et al., 1986; Goodman and Sloviter, 1992; Deller et al., 1994, 1995). In addition, parvalbumin-expressing GABAergic neurons at the granule cell layer/hilar border project contralaterally (Goodman and Sloviter, 1992).

Together these studies led to some question about the location of the mossy cell axon projection—and suggested that mossy cells cannot be defined by a contralateral projection alone. Moreover, it is not possible to define them as a neuron with an inner molecular layer projection. The reason is that CA3 pyramidal cells, mostly in area CA3c and in temporal hippocampus, project to the inner molecular layer (Li et al., 1994).

A greater understanding of the mossy cell axon was made possible by analysis of the axons of intracellularly-labeled hilar cells which had thorny excrescences in the rat (Buckmaster et al., 1996). The labeled cells gave rise to both an ipsilateral and contralateral terminal plexus in the inner molecular layer. Within the ipsilateral projection, the intracellularly-labeled cells had their most extensive projection to the inner molecular layer in distant lamellae relative to the cell body (Figure 3). Interestingly, the projections of intracellularly-labeled mossy cells were not the same. Mossy cells located in the temporal part of the hippocampus projected very far, to distant septal locations. However, mossy cells located in the septal region did not project as far into the temporal hippocampus (Figure 3; Buckmaster et al., 1996). These data were consistent with earlier studies using different methods, which suggested that there was a more extensive spread of the C/A pathway from temporal to septal hippocampus in the mouse (West et al., 1979). Similar results have also been found in the mouse, where calretinin can be used to stain the mossy cells of temporal hippocampus (Blasco-Ibanez and Freund, 1997; Fujise et al., 1998), but in septal hippocampus of the mouse and in rat, calretinin does not stain mossy cells (although calretinin does stain mossy cells in the human; Seress et al., 2008). In mouse, an antibody to calretinin stained the inner molecular layer throughout the septotemporal extent of the DG, even though cell bodies of mossy cells were stained by the antibody in temporal hippocampus only. These data suggested that temporal mossy cells have axons which are highly divergent (Blasco-Ibanez and Freund, 1997).

### Early physiological studies of mossy cells and the questions they raised

The first intracellular recordings from mossy cells were made in hippocampal slices of the guinea pig. These initial studies provided data that led to a reconsideration of the definition of a mossy cell because some cells with dense proximal spines—but not clear thorny excrescences—could not be distinguished from cells with robust thorns. This raised the question: are mossy cells defined by robust thorny excrescences, or is there some variability?

**Figure 4:**
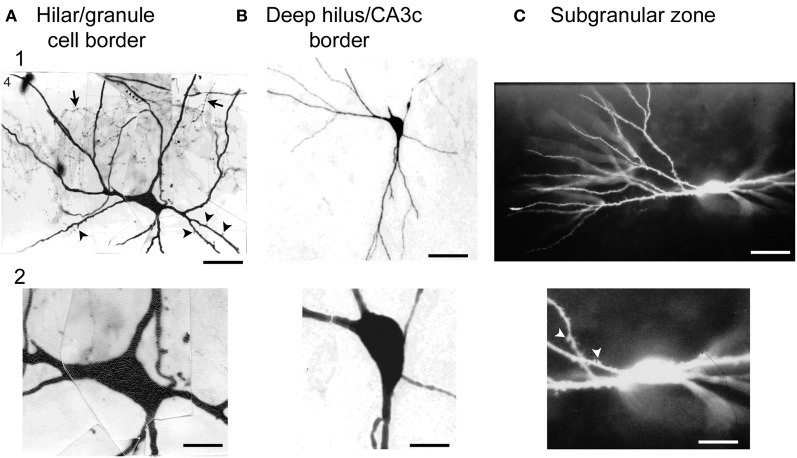
**Large cells of the hilus are not always mossy cells. (A)** A physiologically-identified fast-spiking cell with a basket cell axon, located at the border of the granule cell layer and the hilus. Arrows point to the axon. Arrowheads point to spines. Note the large size of the cell body. Calibration = 30 μm **(A1)**; 15 μm **(A2)**. From Scharfman (1995a). **(B)** A physiologically-identified GABAergic neuron located in the deep hilus near area CA3c with a large cell body. Same calibration as **(A)**. **(C)** A neuron with dense spines all over its dendrites, with physiological characteristics that were not possible to discriminate from mossy cells with large thorny excrescences. This neuron was filled with Lucifer yellow and located on the border of the subgranular zone and the deep hilus. Same calibration as **(A)**. From Scharfman (1993a).

In the recordings, hilar cells were sampled at random with sharp electrodes (i.e., without visualization of the cell before impalement), after physiological properties were characterized, the cell was filled with dye to correlate physiological properties with gross morphological characteristics such as the size of the cell body, and presence of thorny excrescences. The results showed that there was a substantial fraction of neurons in the hilus with physiological properties similar to regular-spiking glutamatergic neurons in other parts of the CNS; the other group of hilar neurons was similar to fast-spiking or slow-spiking GABAergic neurons (below these are all discussed as fast-spiking for simplicity) (Scharfman and Schwartzkroin, 1988; Scharfman, 1992a). The cell bodies of the regular-spiking neurons were usually large and the proximal dendrites were typically large in diameter (relative to granule cells) and covered with thorny excrescences but this was not the case for the fast-spiking cells (Scharfman and Schwartzkroin, 1988). The fast-spiking neurons were generally aspinous, or had spines but they were primarily on dendrites that were not proximal to the soma (Figure 4).

Therefore, there was an apparent division based on physiology and morphology and the regular-spiking neurons appeared to be mossy cells whereas the fast-spiking neurons corresponded to GABAergic neurons. However, the regular-spiking neurons did not always have numerous thorny excrescences (Figure 4). Some of the dendrites merely appeared to be large in diameter and rather “bumpy” which had been shown before (Frotscher et al., 1991). In addition, the somata of the regular-spiking cells were not necessarily larger than the fast-spiking neurons (Figure 4), suggesting that numerous thorny excrescences and a large cell body did not necessarily define mossy cells. The distal dendrites of the regular-spiking neurons were sometimes beaded and lacked spines. These characteristics—beaded, aspiny dendrites—are considered to be common characteristics of interneurons. Therefore, the characteristics of distal dendrites of mossy cells did not seem to be useful in defining them either. Moreover, some fast-spiking neurons had very large (thick) dendrites and spines (Figure 4), suggesting that mossy cells could not be defined by dendrites with a large diameter.

Variations in thorny excrescences in mossy cells also are evident when species are compared. For example, thorny excrescences of hilar cells in the hamster and in humans seem far more “exuberant” than the guinea pig or rat (Murakawa and Kosaka, 2001; Seress et al., 2004; Abraham et al., 2005). Below we argue that exuberant thorny excrescences are not a defining feature of mossy cells, because the cells with robust thorny excrescences (Figure 1C) can not be discriminated from cells with dense proximal spines (Figure 4) using physiological criteria. When the axon is visible, the main branch exits the hilus and enters stratum oriens of CA3, as one would expect for a mossy cells. Therefore, there appears to be some variability in the “moss” and size of mossy cells.

### A proposal for criteria to define mossy cells

Below we suggest the criteria to define a mossy cell based on the data about hilar neurons obtained to date, and based on practical considerations. Each criterion in itself is insufficient to define a mossy cell; together they make a compelling case for a mossy cell.

1. A cell body in the hilus, defined as zone 4 of Amaral (1978).
2. Glutamate as the primary transmitter (other markers are less valuable, as discussed below).
3. An axon that innervates the inner molecular layer.
4. Proximal dendrites with numerous large spines (distal dendrites may be misleading and thorny excrescences are not an absolute requirement).
5. A series of physiological characteristics that distinguish the cell from GABAergic interneurons and CA3 pyramidal cells.

#### Cell body in the hilus

It is hard to argue against the idea that a hilar cell must have a cell body in the hilus. However, the definition of the hilus is not trivial, because the border with CA3c can easily be misconstrued. As shown in Figure 1, Amaral defined the hilus as a specific area between the two blades of the granule cell layer (Amaral, 1978). However, the hilus is not simply the area of the DG that is located between the two blades. Area CA3c (nomenclature of Lorente De Nó, 1934) inserts into the DG, and is not part of the DG (Amaral, 1978). The hilus surrounds the area CA3c cell layer as well as the dendrites of CA3c pyramidal cells; it does not only avoid the cell layer (Amaral, 1978). In the coronal plane of septal hippocampus in rodents, it is difficult to sample neurons from the hilus because area CA3c encompasses the majority of the space between the supra and infrapyramidal blades; there is only a very small area where the hilus is located (Figures 1, 2). In the horizontal plane, the hilus is a larger area (Figures 1, 2).

**Figure 5:**
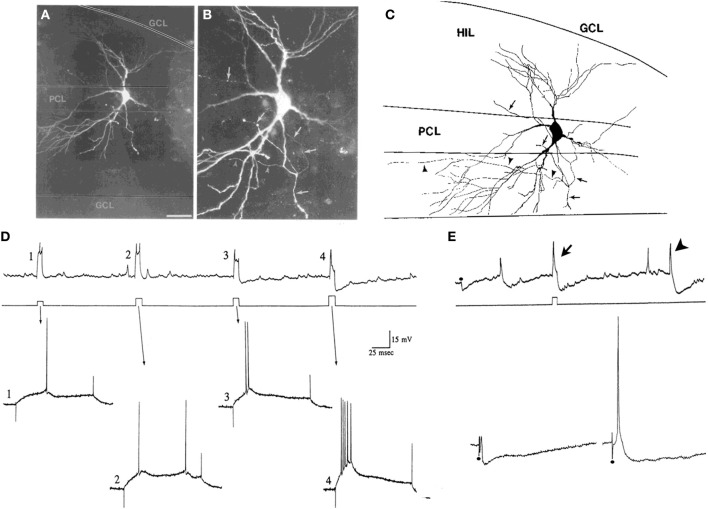
**Characteristics of CA3c “pyramidal cells” and mossy cells. (A,B)** Pyramidal cells of CA3c often have morphology that is not pyramidal-like in that the cell body and proximal dendrites are not pyramidal. They are not interneurons because of their dense spines and physiology. The example shown is from a neuron that was physiologically-identified as a pyramidal cell because of its intrinsic burst firing **(D)**. The merged images through multiple focal planes are shown. Calibration [located in **(A)**] = 100 μm for **(A)** and 20 μm for **(B)**. **(C)** A drawing of the cell in **(A,B)** is shown. Arrowheads indicate the part of the axon that terminated in area CA3. The arrows point to the part of the axon that gave rise to collaterals in the hilus. GCL, granule cell layer; HIL, hilus; PCL, pyramidal cell layer of CA3c. **(D,E)** Physiological discrimination of CA3c neurons from hilar mossy cells. **(D)** A continuous recording from a neuron in CA3c (top) that exhibited firing behavior of a pyramidal cell rather than a mossy cell. During the record, a series of increasing currents are triggered (middle) to elicit firing and the firing behavior is expanded below (arrows). Weak currents (#1–3) did not elicit burst firing but the strongest current command did (#4). The burst in #4 has a characteristic decrement in action potential amplitude, and rides on a triangular depolarization, followed by a large afterhyperpolarization. In contrast, these types of bursts are not found in mossy cells under these recording conditions, and the afterhyperpolarizations are not either. **(E)** CA3c pyramidal cells can also be discriminated from mossy cells by a large IPSP triggered by perforant path or molecular layer stimulation. Top: A continuous record from a CA3c cell showing an IPSP evoked in response to molecular layer stimulation (at the dot) and an afterhyperpolarization following directly-evoked action potentials (arrow). An arrowhead marks a spontaneous burst of action potentials followed by an afterhyperpolarization. Bottom: A response of the same cell to stronger stimuli (at the dots). On the right, the response to the strongest stimulus is shown, which elicited an action potential followed by hyperpolarization, characteristic of pyramidal cells under these recording conditions, but not mossy cells. **(A–E)** are from Scharfman (1993b).

How does one define the border of the hilus with area CA3c? This is relatively straightforward with some staining techniques such as cresyl-violet. Alternatively, almost any stain of the mossy fiber pathway will stain the hilus, but in area CA3c it will only stain stratum lucidum. When there is no staining of the tissue to visualize the CA3c/hilar border, one can identify a cell of interest and then stain the area *posthoc*. Without staining, however, cells near CA3c are difficult to define because cells at the tip of CA3c sometimes appear to be “mossy” but their physiology suggests they are pyramidal cells (Figure 5; Scharfman, 1993b). One criterion that sets these area CA3c cells apart from mossy cells of the hilus is the ability of intracellular current injection to trigger a burst of decrementing action potentials on a triangular depolarization, a typical type of intrinsic firing behavior of CA3 pyramidal cells (Figure 5; Scharfman, 1993b). This is not a characteristic of mossy cells that are close (within 100 μm) to the granule cell layer. When the molecular or granule cell layer is stimulated, an evoked IPSP is a second criterion: in CA3c neurons these IPSPs are robust (Figure 5; Scharfman, 1993a) but this is not the case for mossy cells located close to the granule cell layer. Other characteristics have also been used to distinguish area CA3c pyramidal cells from mossy cells (Buckmaster et al., 1993).

Some of these distinguishing characteristics require close attention to recording conditions. For example, burst firing has been reported in mossy cells of the mouse if the recordings are made in the temporal DG. They occur when ionotropic glutamate receptors and GABA_A_ receptors are blocked (Jinno et al., 2003).

It is important to note that the definition of the hilus, and therefore the hilar/CA3c border, varies with species. In the primate, CA3c is very large and extends very far into the DG. The distance from the granule cell layer/hilar border to CA3c—i.e., the hilus—can be very small.

#### Glutamate as the primary neurotransmitter

Currently mossy cells can be easily distinguished from GABAergic neurons in the hilus because mossy cells are glutamatergic. However, this criterion was not always so clear. One reason to consider that mossy cells might be GABAergic was based on early studies of the inner molecular layer where commissural afferents included type I and type II synapses (Laatsch and Cowan, 1966). Without specific antibodies to glutamate, or direct assessment of monosynaptically-connected neurons, it remained arguable whether mossy cells were a type of GABAergic neuron or were glutamatergic until the early 1990's. Two studies provided evidence that mossy cells were glutamatergic, one anatomical and the second physiological. The first anatomical demonstration of glutamate immunoreactivity was made in Golgi-impregnated mossy cells (Soriano and Frotscher, 1994). The physiological study used hippocampal slices to impale mossy cells—which were confirmed to be regular-spiking, hilar, and had thorny excrescences—and simultaneously recorded from neurons in the granule cell layer until a monosynaptic connection was identified. That study showed for the first time that mossy cells produced unitary EPSPs in granule cells, supporting the hypothesis that mossy cells were glutamatergic (Scharfman, 1995b). Interestingly, the monosynaptic connections between mossy cells and granule cells were weak; they were only detected when a GABA_A_ receptor antagonist was present. In more recent studies using patch recordings and younger tissue, much more robust excitatory connections were evident from mossy cells to GABAergic neurons of the hilus (Larimer and Strowbridge, 2008). One interpretation of these data is that mossy cells may innervate hilar GABAergic neurons close to the mossy cell soma, but preferentially innervate granule cells in distal areas of the hippocampus. Another implication is related to the study of Larimer and Strowbridge, which used young animals (less than 30 days old). Their study suggests that early in life, mossy cells may form a primarily excitatory connection to local GABAergic neurons and this is later refined as their long axon forms synaptic connections to distal granule cells.

Using immunocytochemistry, it is now common to identify mossy cells in the hilus by their immunoreactivity to GluR2/3, a marker of glutamatergic neurons (Leranth et al., 1996). One potential problem, however, is that some granule cells [ectopic granule cells; (Scharfman et al., 2007)] exist in the hilus too. However, they are rare under most conditions, compared to mossy cells (McCloskey et al., 2006; Jiao and Nadler, 2007). Ectopic granule cells arise in greater numbers after pathology. For example, after status epilepticus (SE) in adult rats, GluR2/3- immunoreactive granule cells are common in the hilus (McCloskey et al., 2006; Jiao and Nadler, 2007).

Other markers besides GluR2/3 can be used to identify mossy cells but few are selective. In the mouse, mossy cells in temporal hippocampus express calretinin, as mentioned above. However, there are some hilar GABAergic neurons that express calretinin in rat and mouse (Liu et al., 1996; Martinez et al., 1999) and young granule cells at the granule cell layer/hilar border express calretinin in rats and mice (Brandt et al., 2003; Scharfman et al., 2007). In humans, cocaine- and amphetamine-transcript peptide (CART) stains mossy cells (Seress et al., 2004), but not in rodents. In the rat, one of the glucocorticoid receptors (type II) is present on mossy cells (Patel and Bulloch, 2003). The α8 integrin subunit also stains mossy cells in rats, but not selectively—somatostatin/neuropeptide Y immunoreactive cells stain also (Einheber et al., 2001). Calcitonin-gene-regulated peptide (CGRP)-immunoreactivity is another way to distinguish mossy cells in rat (Bulloch et al., 1996; Freund et al., 1997).

#### An axon that innervates the inner molecular layer

Exceptions to the statement that mossy cells project to the inner molecular layer have not been reported. All neurons with dense proximal spines or thorny excrescences, where an axon has been possible to trace, exhibit an inner molecular layer projection. Therefore, we suggest that one criterion that defines mossy cells is an axon that projects to the inner molecular layer.

However, in some experimental conditions, it is not always possible to determine that an axon is present in the molecular layer. For example, it is hard to find a mossy cell axon in the inner molecular layer in most hippocampal slices. In these slices, however, it is often possible to trace the major branch of the axon to its point of exit from the DG in stratum oriens of area CA3b/c. It can be distinguished from axon collaterals by its large diameter, and the fact that most axon collaterals are restricted to the hilus. In contrast to mossy cells, other hilar cell types do not have this axon projection, and neurons in area CA3c do not either, making it a practical method to differentiate mossy cells in hippocampal slices from other cell types.

It is not widely appreciated that the mossy cell also has a dense local axon collateral plexus in the hilus, which was emphasized in early studies of mossy cells (Frotscher et al., 1991). In recordings of mossy cells in hippocampal slices, there is a dense network of hilar collaterals (Scharfman and Schwartzkroin, 1988; Buckmaster et al., 1992; Larimer and Strowbridge, 2008), which have implications for the potential functional role of mossy cells. The local axon collaterals are depicted schematically in Figure 3 and discussed further below.

Although it is often hard to find the branches of the local axon that extend into the inner molecular layer in slices, empirical evidence suggests they are present. Thus, the mossy cell axon that ends in the inner molecular layer was found after injecting dye into mossy cells in hippocampal slices which were only 400 μm thick (Buckmaster et al., 1992). Furthermore, effects of mossy cells on granule cells have been reported in 400 μm-thick hippocampal slices (Scharfman, 1995b; Jackson and Scharfman, 1996). Changes in physiology of the DG in hippocampal slices have also been detected before and after selective ablation of mossy cells (Ratzliff et al., 2004).

Regarding other inputs to the inner molecular layer besides mossy cells, there is a long list of inputs that are notable. CA3c pyramidal cells that are located in the temporal pole of the hippocampus project to the inner molecular layer (Li et al., 1994). The supramammillary nucleus sends projections to the border of the inner molecular layer and the granule cell layer (Leranth and Hajszan, 2007). Hilar GABAergic neurons provide GABAergic input [HICAP cells; (Han et al., 1993)]. Diverse brainstem nuclei (dorsal raphe, locus coeruleus) project to the molecular layer, including the inner molecular layer (Amaral and Campbell, 1986; Swanson et al., 1987).

#### Proximal dendrites with numerous spines

Based on the inability to discriminate physiological differences between cells with obvious thorny excrescences (Figure 1C) and cells with dense proximal spines but thorny excrescences that are not as clear (Figure 4C), it seems reasonable to suggest that mossy cells are characterized by large proximal spines, whether or not they can be called thorny excrescences. This is practically useful because the discrimination between dense spines and small thorny excrescences is somewhat subjective, in our view. The proximal dendrites are the most important area to consider in this assessment because distal dendrites of mossy cells may have spines that are far less robust, and some interneurons have distal dendrites where spines are robust.

#### Physiological characteristics that distinguish mossy cells from other cell types

***Intrinsic properties.*** Mossy cells that were initially characterized in guinea pig slices, and subsequently in slices of rat and other species, have “regular-spiking” physiology. Classically the term “regular-spiking” refers to the width (duration) of the action potential. The longer duration of the action potential of mossy cells compared to GABAergic neurons is very easy to discriminate, whether the action potential is triggered by direct current, it occurs spontaneously, or it occurs in response to synaptic stimulation.

However, there are a few potential problems with the implementation of this criterion. One is the fact that almost any cell, if unhealthy, develops a broader action potential. And, in slices, the vulnerability of mossy cells to trauma appears to make them unhealthy unless great care is taken to prepare the slices. Therefore, other criteria are useful. For example, an additional characteristic that is useful to discriminate mossy cells is the ratio of the rate of rise to the rate of decay of the action potential. The ratio is much greater than the one for mossy cells and pyramidal cells but approximates one for GABAergic neurons (Scharfman, 1993b, 1995a).

**Figure 6:**
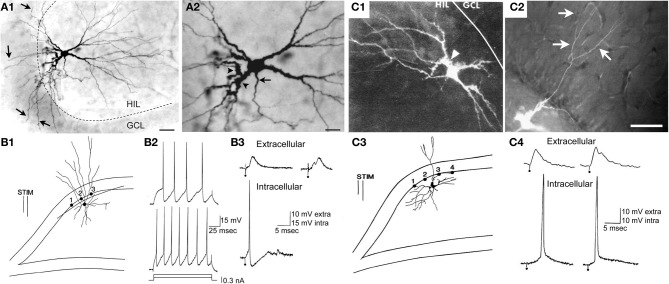
**A subset of hilar cells have low thresholds in response to electrical stimulation of the perforant path in rat hippocampal slices. (A1)** Mossy cells often have dendrites in the molecular layer if their cell body is located near the granule cell layer. A Neurobiotin-filled physiologically-identified mossy cell is shown as an example. There are numerous dendrites entering the granule cell layer (GCL) and molecular layer (arrows). The dotted line marks the border of the HIL and GCL. Calibration = 80 μm. **(A2)** The same cell is shown at higher magnification. The arrow indicates the axon; arrowheads mark thorny excrescences. More examples are shown in Scharfman (1991). Calibration = 40 μm. **(A)** is from Scharfman et al. (2001). **(B1)** A drawing of a hilar interneuron with a low threshold is shown. **(B1–B3)** Are sites where the response to electrical stimulation of the molecular layer was recorded to evaluate granule cell responses to the same stimulus. The bipolar stimulating electrode is indicated by two parallel lines (STIM). **(B2)** Intracellular current (0.15, 0.3 nA) was used to evaluate firing behavior, and the responses demonstrated typical firing of GABAergic neurons: weak spike frequency adaptation. **(B3)** Top (extracellular): the response recorded extracellularly at site #1 at weak (left) and strong (right) intensities of stimulation. Bottom (intracellular): simultaneously recording of the response to the weak stimulus in the interneuron shown in **(B1)** The interneuron reached threshold but there was no indication of suprathreshold activation of granule cells at the same stimulus strength. At the higher intensity of stimulation, a population spike occurred, signaling firing in granule cells. Calibration: 10 mV, extracellular; 15 mV, intracellular. **(C1,C2)** A mossy cell with a molecular layer dendrite is shown after filling the cell with Lucifer yellow in a rat hippocampal slice. This dendrite bifurcated after exiting the granule cell layer and reached the outer molecular layer. In the outer two-thirds of the molecular layer there were additional branches (arrows in **C2**). The arrowhead **(C1)** marks a thorny excrescence. Calibration = 100 μm. **(C3)** A drawing of the cell shown in **(C1,C2)**. **(C4)** Simultaneous extracellular recording from the granule cell layer at the site closest to the stimulating electrode in **(C3)** and intracellular recording from the mossy cell shows a lower threshold for action potential generation in the mossy cell compared to the field potential. It is important to note that it might only take 1 granule cell action potential to activate the mossy cell because of the large quantal size of a granule cell unitary EPSP in a mossy cell (Scharfman et al., 1990) and this might not be reflected in the field potential, which is an average of many cells located only near the recording electrode. Therefore, many locations were sampled, especially those near the stimulating electrode where a granule cell might be directly activated, before concluding that the mossy cell had a relatively low threshold compared to adjacent granule cells. **(B–C)** are from Scharfman (1991).

Additional physiological characteristics of mossy cells in slices distinguished them from other cell types. For example, mossy cells have very long time constants (>20 ms in the guinea pig or rat) which are similar to CA3c pyramidal cells. In contrast, granule cells and interneurons have relatively short time constants (<15 ms). The absolute numbers may vary depending on the recording method (sharp or patch) but the relative differences remain, making this criterion very useful. Mossy cells also have a very small afterhyperpolarization (AHP) following an action potential compared to GABAergic neurons. Interneurons have large AHPs and typically have much less variability in the AHP from one command pulse to the next, and have much less adaptation, than mossy cells [Figure 6 (Scharfman, 1992a, 1995a; Buhl et al., 1994; Lübke et al., 1998)].

The firing behavior of a mossy cell seems easier to distinguish from other hilar neurons using a sharp electrode than a patch electrode (Lübke et al., 1998), probably because a patch electrode has constituents that affect firing substantially and if the same internal solution for the patch electrode is used across cells, the more the firing of different cells is similar.

***Synaptic responses.*** One characteristic of mossy cells is a large, frequent barrage of spontaneous synaptic input (Scharfman and Schwartzkroin, 1988; Strowbridge et al., 1992; Scharfman, 1993a; Soltesz and Mody, 1994). At resting potential, this is evident as depolarizations which can trigger action potentials, which are blocked by excitatory amino acid receptor antagonists, so they are EPSPs. Presumably the majority of the input is due to spontaneous release from mossy fiber boutons, but mossy cells also receive local input from CA3 pyramidal cells, and cut axons of extrinsic inputs to the hilus may release transmitter also. Mossy cells are innervated by GABAergic cells, and when glutamate receptors are blocked, inhibitory potentials or IPSCs are readily detected (Scharfman, 1992b; Soltesz and Mody, 1994). In contrast, hilar interneurons have spontaneous input that is much smaller and has faster kinetics (Scharfman et al., 1990; Livsey and Vicini, 1992).

When stimulating electrodes are used to evaluate synaptic inputs to hilar neurons, EPSPs and IPSPs can be evoked by perforant path stimulation in almost all cells. There is extensive variation within a given hilar cell type (Scharfman, 1995a), and as a result, this information—while very important—does not clarify the hilar cell type easily.

## Are there subtypes of mossy cells?

In light of the variability in many of the characteristics of mossy cells, one might suggest that there could be subtypes of mossy cells. For example, one subtype has dense spines on their proximal dendrites, but no thorny excrescences, and another subtype might have thorny excrescences. This division would certainly be possible to make, but does not seem useful, because physiological or functional distinctions are not evident when comparing cells with dense proximal spines and cells with thorny excrescences.

**Figure 7:**
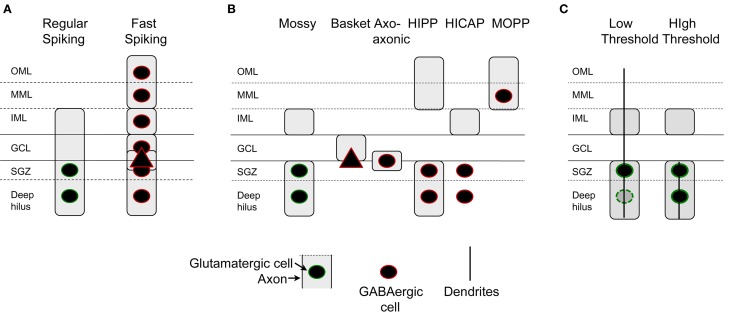
**Characterization of dentate gyrus non-granule cells. (A)** Intrinsic electrophysiology of DG non-granule cells include two classes primarily, those cells that are regular-spiking and those that are not. Regular-spiking cells are mossy cells with hilar cell bodies and an axon in all layers but the outer and middle molecular layers. Fast-spiking cells are found in all layers and have axons that are in all layers. **(B)** Axon location indicates at least six types of non-granule cells. GABAergic neurons include basket cells, typically with a pyramidal cell-shaped soma, axo-axonic cells, HIPP cells, HICAP cells, and MOPP cells (Han et al., 1993; Freund and Buzsáki, 1996). Recent studies suggest that neurogliaform cells and ivy cells exist (Armstrong et al., 2011, 2012). **(C)** Mossy cells can be divided into two categories based on the location of their dendrites, cell body, and threshold to stimulation of the perforant path. Cells with dendrites in the molecular layer and primarily located near the cell layer have low thresholds for activation by the perforant path.

However, there is one division that seems useful, because it is detectable anatomically and also physiologically: some of the mossy cells recorded in rat hippocampal slices have a low threshold for action potential generation when the perforant path is stimulated electrically in slices, compared to granule cells located nearby (Scharfman, 1991). Thus, perforant path stimulation can evoke action potentials in these “low threshold” mossy cells before the stimulus is increased sufficiently to elicit a detectable population spike in the granule cells next to it, or action potentials in a granule cell selected at random from that population. The mossy cells with low thresholds are usually located close to the granule cell layer, and have a relatively thin dendrite that passes into the molecular layer and can either stop in the inner or middle molecular layer or extend to the hippocampal fissure (Scharfman, 1991; Figure 6). In contrast, mossy cells without these dendrites appear to have a threshold similar to or higher than granule cells in the same slice and are activated at a latency consistent with a perforant path-to-granule cell-to-mossy cell pathway (i.e., disynaptic). Therefore, it seems reasonable to suggest that mossy cells with low thresholds are a subtype of mossy cell (Figure 7). There also are hilar GABAergic interneurons with dendrites in the molecular layer and low thresholds to perforant path stimulation in hippocampal slices [Figure 6 (Scharfman, 1991)], which could be innervated by perforant path fibers that innervate hilar GABAergic dendrites (Deller et al., 1996) or perforant path innervation of molecular layer dendrites [Figure 6 (Scharfman, 1991)]. These GABAergic hilar neurons with low thresholds and both molecular layer and hilar dendrites may correspond to HIPP cells which co-express somatostatin and neuropeptide Y (see Figure 7).

The pathway that causes the short latency, low threshold activation of hilar cells with molecular layer dendrites could be the perforant path, based on the demonstration that perforant path fibers in the molecular layer innervate GABAergic neurons. There is no anatomical evidence that the perforant path innervates mossy cell dendrites in the molecular layer, however. One study that could be relevant showed that deep layer entorhinal neurons have axons that enter the inner molecular layer, granule cell layer, and hilus (Deller et al., 1996). That study suggested that the axons innervated GABAergic neurons, not mossy cells. However, some axons terminated on spines, so it is possible mossy cell dendrites were contacted. In summary, both mossy cells and hilar GABAergic neurons with dendrites in the molecular layer are neurons that appear to have low thresholds. The reason for their low thresholds could be direct innervation by the perforant path, but this explanation is not as well developed for mossy cells as it is for GABAergic neurons.

Notably, the neurons with low thresholds are a subset of mossy cells in the normal rat, but appear to be rare in some species, such as the mouse (Kowalski et al., 2009). In the primate, mossy cells with molecular layer dendrites and even CA3c pyramidal cells with molecular layer dendrites have been shown [the “dentate-CA3 cell”; (Buckmaster and Amaral, 2001)]. The numbers of these cells relative to the entire population of mossy cells and CA3c pyramidal cells is not clear, but it seems likely that they are a minority. In contrast, the reeler mouse is vastly different (Kowalski et al., 2009), where disorganization of the cell layers of the DG is accompanied by many mossy cell dendrites in the molecular layer, which are innervated by the perforant path (Kowalski et al., 2009).

## Vulnerability of mossy cells

The vulnerability of the hilar region or “endfolium” in humans has been known for some time. One of the first studies to suggest that hilar neurons might be vulnerable relative to other hippocampal cell types was a study of postmortem brain samples from patients with temporal lobe epilepsy (TLE). It was noted that endfolium sclerosis, where only hilar neuron loss occurs, was often evident. In addition, there was often damage in other hippocampal subfields such as CA1 and CA3 (Margerison and Corsellis, 1966). The results suggested that hilar neuron loss might be a “common denominator” in TLE, and led to the hypothesis that hilar neuron loss might cause TLE.

In 1987 a study was published that attempted to simulate endfolium sclerosis in rats by prolonged activation of DG granule cells by intermittent perforant path stimulation for 24 h (Sloviter, 1987). It was shown that hilar neurons which lacked GABA immunoreactivity (presumably mossy cells) were vulnerable. This was notable because a leading hypothesis for epilepsy at the time was a loss of GABAergic neurons—not loss of glutamatergic neurons (Ribak et al., 1979).

Sloviter and colleagues showed using silver stain that terminals in the inner molecular layer were degenerated after prolonged stimulation, which also suggested that mossy cells were damaged. Together with earlier studies (Olney et al., 1983; Sloviter, 1983) and consistent with other ideas at the time (Mattson et al., 1989), it was hypothesized that release of glutamate from the large boutons of granule cells was excitotoxic to hilar cells such as mossy cells (Olney et al., 1986).

As animal models of TLE were developed, investigators began to study neuronal loss in the hilus after insults and injury that are risk factors for TLE, including a brief period of severe continuous seizures (SE). Hilar neuron loss was documented in these animals, and included both mossy cells and HIPP cells (Maglóczky and Freund, 1993, 1995; Mitchell et al., 1995, 1997), which was later studied in more detail by others (Buckmaster and Jongen-Relo, 1999; Sun et al., 2007). After hypoxia/ischemia, mossy cells and HIPP cells were also reduced in number (Johansen et al., 1987; Crain et al., 1988; Represa et al., 1991; Hsu and Buzsáki, 1993; Matsuyama et al., 1993). After fluid-percussive injury, a model of traumatic brain injury, a large reduction in mossy cells and HIPP cells occurred (Lowenstein et al., 1992; Santhakumar et al., 2000). In all of these conditions, granule cells were spared, suggesting a selective vulnerability of hilar mossy cells and HIPP cells.

Many questions were raised by the results. What was the normal role of mossy cells and HIPP cells? Did mossy cell and HIPP cell loss cause TLE? To date there is no method to selectively remove mossy cells or HIPP cells or to silence them, so investigators have used the animal models where they are reduced in number to try to gain insight into these questions.

## Understanding mossy cells by examining their vulnerability

One hypothesis for the vulnerability of hilar mossy cells and HIPP neurons was based on the fact that markers of calcium binding proteins did not stain mossy cells and HIPP cells. Two calcium binding proteins were investigated primarily: parvalbumin, a marker of relatively resistant perisomatic targeting GABAergic cells, and calbindin D28K (CaBP), which primarily stains granule cells within the DG. The correlation between staining for these two calcium binding proteins and relative resistance to injury, taken together with the idea that excitotoxicity was caused by calcium accumulation, led to the hypothesis that calcium binding capacity was strong in resistant neurons and weak in vulnerable neurons (Sloviter, 1989). Therefore, it was suggested that mossy cells and HIPP cells were vulnerable because they lacked calcium binding capacity. When granule cell input was strong, excitotoxicity occurred more readily than in other cell types, because intracellular calcium buffering was limited. In support of that hypothesis, intracellular calcium chelation by a synthetic calcium chelator, BAPTA, led to resistance of hilar mossy cells and hilar interneurons to prolonged perforant path stimulation in rat hippocampal slices (Scharfman and Schwartzkroin, 1989). This hypothesis was also supported by anatomical studies showing that the relatively resistant CA2 region expresses CaBP (Leranth and Ribak, 1991). Moreover, we found that CaBP expression occurred in surviving hilar neurons after SE, suggesting that those hilar cells which survive might express CaBP *de novo* as an endogenous mechanism for protection (Scharfman et al., 2002b; Scharfman, 2012a). However, it has not been proved, to our knowledge, that calcium binding proteins are the reason for vulnerability or resistance. In fact, exceptions to the correlation between CaBP and parvalbumin expression have been described, which argued against the hypothesis (Freund et al., 1990, 1992; Bouilleret et al., 2000).

Another hypothesis for the vulnerability of hilar mossy cells is the nature of their mossy fiber input (Schwartzkroin et al., 1996). It appears that mossy cells receive more of the “massive” mossy fiber boutons—relative to the smaller boutons of mossy fibers—than the GABAergic hilar cells and CA3 pyramidal cells, although quantitative comparisons are unavailable. Also, the large mossy fiber boutons are proximal to the soma of mossy cells where they are likely to have the most impact. It is clear when recording from mossy cells that they receive a great deal of excitatory drive, because there is usually a continuous barrage of depolarizing input in the form of EPSPs. This barrage may indeed place the cells at risk of excitotoxicity during injury, because when slices are made without a great deal of care, the mossy cells with the greatest frequency of these spontaneous EPSPs are harder to detect compared to slices with more attention to preservation of the hippocampus. This observation—albeit an anecdotal one—suggests that the mossy cells with the greatest spontaneous activity did not survive the trauma of slice preparation but mossy cells with less spontaneous input did.

Another regulator of vulnerability, which has been examined mostly in HIPP cells, is expression of striatal enriched protein tyrosine phosphatase; STEP) (Choi et al., 2007). HIPP cells have low expression, and other hilar cells (possibly mossy cells) appear to also exhibit low expression based on the micrographs that are published, whereas some large cells near the HIPP cells in the hilus also have low expression of STEP (Choi et al., 2007). When challenged with an insult, STEP can rescue HIPP cells (Choi et al., 2007). HIPP cells are also vulnerable in response to degeneration of the septohippocampal projection (Zhang et al., 1998), and are reduced in number in postmortem specimens of individuals with Alzheimer's disease (Chan-Palay, 1987); vulnerability in Alzheimer's disease may also be the case for mossy cells but less evidence is published for mossy cells compared to HIPP cells (Scharfman, 2012a).

## Understanding the functional role of mossy cells

### Inferences based on the consequences of mossy cell loss

#### The dormant basket cell hypothesis

**Figure 8:**
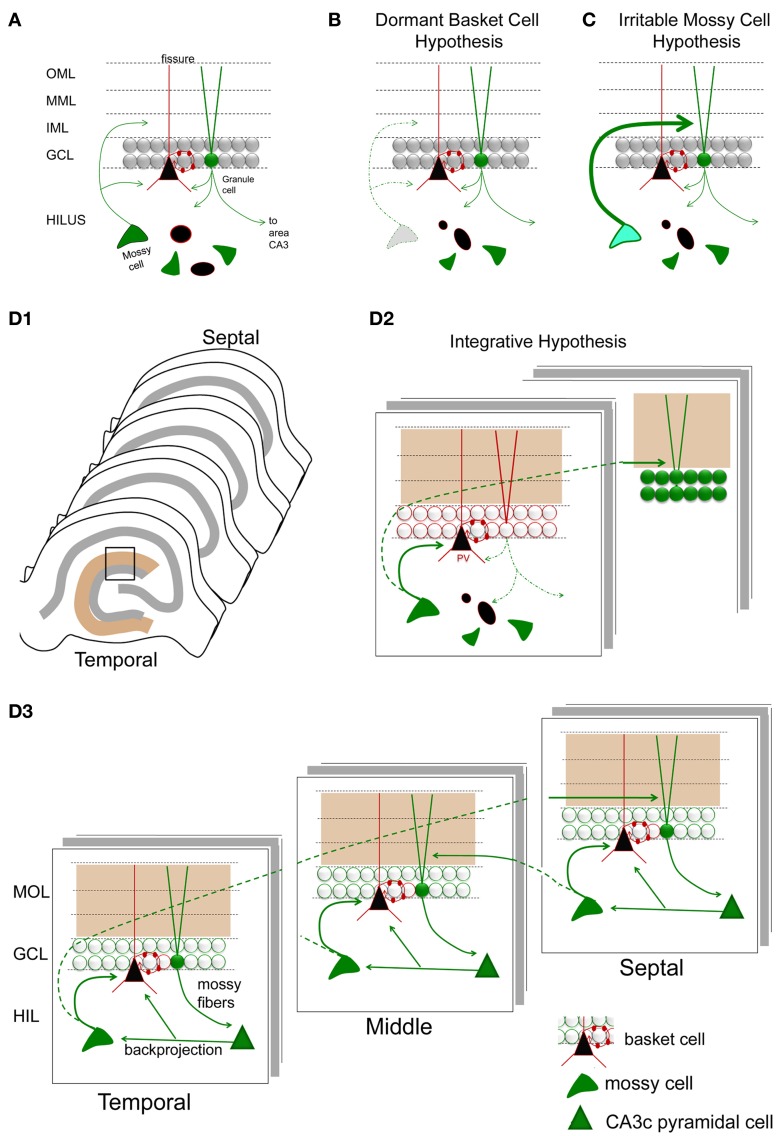
**Hypotheses for mossy cell vulnerability and function. (A)** An illustration of the circuit components used in parts **(B–D)**. Only some cell types in the DG are shown. Green cells and fibers represent glutamatergic cells and their axons; black cells and their red processes represent GABAergic neurons and their dendrites/axons. The triangular black cell represents the prototype of the GABAergic neuron, the basket cell. **(B)** The dormant basket cell hypothesis is illustrated schematically. Without mossy cell afferent input, basket cells do not have sufficient afferent input to inhibit granule cells. The result is disinhibition of granule cells. **(C)** The irritable mossy cell hypothesis is illustrated schematically. When mossy cells are activated, they directly excite granule cells. After traumatic brain injury, they discharge more. The net effect is more granule cell excitation. **(D1)** A representation of the lamellae of the hippocampus is shown. Brown = molecular layer. Gray = cell layers. **(D2)** A hypothesis that incorporates aspects of the dormant basket cell and irritable mossy cell hypotheses. (a) Mossy cell axons near their cell body, i.e., within the lamella of the mossy cell soma, innervate GABAergic neurons primarily, leading to inhibition of adjacent granule cells; (b) in distant lamella, the same mossy cells primarily excite granule cells. **(D3)** A schematic that illustrates a modification of the hypothesis of Lisman et al. (2005) to account for differences in circuitry across the septohippocampal axis, and GABAergic inhibition by the backprojecting axon collaterals of CA3 pyramidal cells. Following granule cell activation by the perforant path, CA3 activation by the mossy fibers will be followed by excitation of GABAergic neurons in the DG by CA3 backprojections [including the hilar dendrites of basket cells as shown; (Kneisler and Dingledine, 1995)]. The backprojection also innervates mossy cells which excite local GABAergic neurons. The result is silencing of recently active granule cells. In distal hippocampus, granule cells will be stimulated by the same mossy cells. CA3 backprojections in that location will then activate mossy cells which will project back to a lamella close to where activity began. This lamella is unlikely to be precisely the same as the original one because the extent that temporal mossy cells project to septal hippocampus is greater than the extent that septal mossy cells project to temporal levels. Note that the circuitry is simplified in the schematic for the purposes of illustration.

Based on the apparent loss of hilar mossy cells, and preservation of GABAergic basket cells after prolonged perforant path stimulation in the rat, it was proposed that hyperexcitability following mossy cell loss was caused by inadequate activation of basket cells by mossy cells (Sloviter, 1991). Basket cells are a major subtype of interneuron in the DG, which play a major role in perisomatic inhibition of granule cells. Without mossy cell input, the basket cells were suggested to be “dormant”—fully functional, but lacking a major source of afferent input (Figure 8). Evidence for this hypothesis was provided by several studies, each consistent with the idea that basket cells were present and functional, but lacked their normal excitatory input (Sloviter, 1991, 1994). The idea of dormant basket cells was also applied to other circuits (Bekenstein and Lothman, 1993).

Since that time, the dormant basket cell hypothesis has been questioned. One reason is that loss of HIPP cells might also cause hyperexcitability. Another reason is that granule cells provide strong afferent input to basket cells and are resistant to insult and injury. Also, additional data have suggested that postsynaptic changes in GABAergic synapses on granule cells occur under conditions of mossy cell loss (Brooks-Kayal et al., 1998; Mtchedlishvili et al., 2001; Zhang and Buckmaster, 2009) so presynaptic mechanisms are not necessary to invoke. The idea that the basket cell is the major cell type that controls granule cell excitability has also been modified; it is now clear that other subtypes of GABAergic interneurons are very important (Freund and Buzsáki, 1996). Therefore, the “dormant” basket cell hypothesis has several potential limitations (Bernard et al., 1998). An alternative to the dormant basket cell hypothesis, for example, is a relatively recent idea that NaV1.1 sodium channels of DG basket cells are altered in epilepsy or Alzheimer's disease, but afferent input is unchanged. For example, in mouse models of familial Alzheimer's disease, it has been suggested that Nav1.1 sodium channels are reduced at the cell surface of GABAergic basket cells of the DG, leading to disinhibition of granule cells; in some genetic forms of epilepsy (Generalized epilepsy with febrile seizures-plus; Severe myoclonic epilepsy in infancy), mutations in Nav1.1 cause the disease (Catterall et al., 2010; Scharfman, 2012b; Verret et al., 2012).

#### Irritable mossy cell hypothesis

Vulnerability of mossy cells was also addressed by detailed studies of the fluid-percussive injury model of traumatic brain injury in rats. In this animal model, it was shown that mossy cell loss occurred 1 week following injury, and mossy cell loss was not necessarily greater than the loss of GABAergic neurons (Santhakumar et al., 2000). The relative loss of mossy cells vs. GABAergic neurons was hard to address because—as the authors noted—injury changes expression levels of proteins used to identify GABAergic neurons. The authors conducted mRNA expression studies to support their findings from immunocytochemistry, although even mRNA measurements have caveats, as the authors pointed out. Nevertheless, it was clear that there were surviving mossy cells after brain injury, and granule cell hyperexcitability developed. Interestingly, the hyperexcitability of granule cells resembled the findings of the prolonged perforant path stimulation model, i.e., a stimulus to the perforant path evoked a short train of 2–4 population spikes of granule cells in the injured animals. The results were also similar to other studies of fluid percussion injury where destruction of hilar cells and a short train of 2–4 population spikes were recorded in response to stimulation of the perforant path (Lowenstein et al., 1992). Importantly, surviving mossy cells were more active in slices from the injured animals, and it was suggested that this mossy cell hyperexcitability could directly cause the granule cell hyperexcitability in distal hippocampus (Figure 8). It was proposed that surviving mossy cells became “irritable” (more excitable) after injury, and contributed to hyperexcitability of distal granule cells as a result (Santhakumar et al., 2000, 2005; Ratzliff et al., 2002).

The irritable mossy cell hypothesis is important in light of recent approaches to animal models of epilepsy where the use of SE to induce hippocampal injury has been modified. Instead of producing complete hilar loss by prolonged SE, a less severe SE is induced which produces less damage and less hilar loss (Scharfman et al., 2001, 2002a, 2009). SE severity is reduced by administration of an anticonvulsant (e.g., diazepam) ≤1 h after seizures begin. The result is less damage to hilar mossy cells, hilar GABAergic neurons, and the rest of the brain (Scharfman et al., 2001, 2002a).

Animals that were examined that experienced SE with reduced severity showed effects that supported the irritable mossy cell hypothesis. Surviving mossy cells exhibited spontaneous burst discharges called paroxysmal depolarization shifts (Scharfman et al., 2001), the hallmark behavior of epileptic cortical principal cells (Prince, 1968; Ayala, 1983). A subset of additional hilar cells which were fast-spiking, and therefore GABAergic neurons, also exhibited these discharges (Scharfman et al., 2001). The generator of the epileptiform activity was area CA3, and activity reached the hilus by the backprojecting CA3 axon collaterals (Scharfman, 2007). Thus, severing the junction between the DG and CA3 silenced the DG mossy cells (Scharfman, 1994b). The results suggested that, in both an animal model of traumatic injury and an animal model of epilepsy, mossy cells did not necessarily die, and the surviving mossy cells became hyperexcitable.

Based on these two hypotheses, the dormant basket cell hypothesis and the irritable mossy cell hypothesis (Figure 8), there are two major concepts that have developed to explain the functional role of mossy cells in the DG. First, mossy cells are critical for inhibition of granule cells because of their excitatory effect on GABAergic neurons, which in turn inhibit granule cells. Second, mossy cells have a potentially powerful direct excitatory role on distal granule cells. How does one reconcile these two ideas?

### An integrative hypothesis for mossy cell function

Both hypotheses may be correct. Local to the cell body of the MC, there is a local plexus of mossy cell axon collaterals that could be primarily inhibitory; this idea is supported by the results showing that mossy cells have monosynaptic excitatory connections with local inhibitory neurons in hippocampal slices using paired recordings (Scharfman, 1995b; Larimer and Strowbridge, 2008). If GABA_A_ receptors are blocked, monosynaptic excitatory connections of mossy cells to granule cells can be detected (Scharfman, 1995b), suggesting that normally GABAergic inhibition masked excitatory effects of mossy cells on granule cells. However, other studies have shown that excitatory actions of MCs can be detected in hippocampal slices even when GABA_A_ receptors are not blocked (Jackson and Scharfman, 1996).

Distal to the area where the mossy cell body is located, the mossy cell axon appears to primarily innervate granule cells. This idea is supported by quantitative studies of the mossy cell projection distal to the location of the cell body. At these distal locations the mossy cell axon preferentially innervates granule cells compared to GABAergic neurons (Buckmaster et al., 1996).

Based on these data, the following circuitry is suggested (Figure 8D): upon activation of a granule cell by entorhinal cortex, local inhibition of granule cells by mossy cells limits the activation of the recently-activated granule cell. Reducing the activation of these granule cells may be important for functions related to pattern separation, where it is not ideal for granule cells to discharge persistently. When pattern separation is simulated by a computational model of the DG network, removal of mossy cells indeed degrades the ability of the network to distinguish a set of overlapping input patterns (Myers and Scharfman, 2009, 2011). In addition to local inhibition of granule cells that were recently active, mossy cells could be important to activate distal granule cells which were not activated by the initial entorhinal input. Therefore, local inhibition and distal excitation of granule cells by mossy cells could be an effective modulation of the DG network to promote pattern separation by granule cells.

This hypothesis is complementary to one that was proposed before that suggests the DG and mossy cells are important to associative memory (Buckmaster and Schwartzkroin, 1994). It is also complementary to the idea that mossy cells are critical to the ability of the hippocampus to learn sequences of information (Lisman et al., 2005). In the model proposed by Lisman et al. (2005), area CA3 is an autoassociative network and the DG is heteroassociative; the granule cells and CA3 interact to perform the task of sequence learning and sequence prediction. In this DG-CA3 network, input from the DG is provided to the CA3 autoassociator, which performs pattern completion along recurrent collaterals among pyramidal cells; the resulting pattern is provided back to DG via the CA3 backprojection; and the DG then performs heteroassociation to predict the next inputs which will arrive from entorhinal cortex. Together, DG and CA3 can learn and reproduce sequences of patterns via this reciprocal loop. Mossy cells play a critical role in this network in two ways. First, mossy cells mediate excitatory input from CA3 to granule cells (the pathway is CA3 pyramidal cell-to-mossy cell-to-granule cell). Second, mossy cells themselves can mediate an additional heteroassociative pathway (as proposed by Lisman et al., 2005) because mossy cells form a second reciprocal loop with granule cells.

Although the models of Myers and Scharfman (2011), which stress granule cell inhibition, and of Lisman et al. (2005), which stress granule cell excitation, appear opposing, the three dimensional structure of the hippocampus may provide a way to reconcile these ideas, diagrammed in Figure 8D. Specifically, it has been shown that the CA3 backprojection innervates both GABAergic interneurons and mossy cells. In hippocampal slices, normally the inhibition dominates, probably because most of the axon projection of mossy cells that excite granule cells is transected (Scharfman, 1994a,b). However, this may not be the case *in vivo*. Rather, in a given lamella, area CA3 excites mossy cells which in turn activate distal granule cells, and at the same time CA3 excites GABAergic neurons which inhibit granule cells within the lamella. Inhibition of granule cells within the lamella could potentially promote pattern separation as proposed by Myers and Scharfman (2011), while activation of distal granule cells by mossy cells could provide the heteroassociative component of the Lisman et al. (2005) model. These distal granule cells would update area CA3 neurons in the same distal part of hippocampus because mossy fiber axons of granule cells are a lamellar pathway. Thus, a focus on the three-dimensional structure of DG-CA3 circuitry may promote understanding of multiple functions within the same substrate.

### An explanation for mossy cell vulnerability based on their normal role in the dentate gyrus

A corollary to this hypothesis is that mossy cells are relays to granule cells, and their high sensitivity is important to the activation of otherwise silent granule cells. This function may be critical and therefore worth the “price” of a high risk of excitotoxicity to mossy cells.

One reason to suggest that mossy cells are relays is based on their afferent inputs relative to granule cells. Mossy cells receive numerous intrinsic and extrinsic inputs which do not innervate granule cells. For example, CA3 pyramidal cells innervate mossy cells throughout a large part of the septohippocampal axis, but only in temporal hippocampus does CA3 innervate granule cells (Li et al., 1994). The fact that mossy cells are the only dentate neuron with the glucocorticoid receptor subtype 2 receptor (Patel and Bulloch, 2003) suggests a potential role in stress that is absent in granule cells. There also is a great deal of extrinsic subcortical input to the hilus, and in some cases, the input to the hilus appears to be greater than the molecular layer, suggesting that the input has a greater effect on hilar neurons than granule cells (Amaral and Campbell, 1986; Swanson et al., 1987). For example, serotoninergic fibers are dense in the subgranular zone compared to the molecular layer (Swanson et al., 1987). Dopaminergic fibers and norardrenergic fibers also appear to innervate the hilus more than the molecular layer (Swanson et al., 1987). However, there is some disagreement about the relative patterns of inputs from these ascending brainstem systems, possibly due to species differences and differences among the antibodies that have been employed (compare Amaral and Campbell, 1986 with Swanson et al., 1987). It is also important to be cautious in the interpretation of function based on immunocytochemistry, because the pharmacological effects of the transmitters in these fiber pathways are likely to be complex given the large numbers of receptor subtypes.

Mossy cells are well designed to serve as a relay cell for other reasons. For example, mossy cells have a resting potential close to threshold and receive a constant barrage of mossy fiber input that keeps them even closer to threshold (Scharfman and Schwartzkroin, 1988; Scharfman, 1993a). Mossy cells also have a characteristic that may be important if they are a relay cell to granule cell, because the characteristic predisposes them not to activate granule cells too much: if mossy cells are induced to fire repetitively in response to a strong glutamatergic or direct stimulus, their ability to discharge decays. Their action potential broadens, shortens, and ultimately they stop firing (Scharfman and Schwartzkroin, 1989, 1990). On the other hand, it has been reported that the reverse is true under some conditions (Strowbridge et al., 1992), which suggests a potential for plasticity of relay function.

There is one “problem” with the idea that mossy cells are important as a relay of information to granule cells: the unitary EPSP of mossy cells to granule cells seems quite weak and often fails (Scharfman, 1995b). However, when granule cells are depolarized, and potentially there is also a decrease in GABAergic inhibition of the granule cells, mossy cell input strengthens (Scharfman, 1995b). Therefore, if a septal GABAergic input (for example) disinhibits the granule cells, which appears to be its primary function in the DG (Freund and Antal, 1988), it may also disinhibit a mossy cell, making distal granule cells more likely to be activated by input from mossy cells. Another combination that would be potent is noradrenergic depolarization of granule cells (Lacaille and Harley, 1985; Lacaille and Schwartzkroin, 1988; Dahl and Sarvey, 1989) at the same time as a mossy cell input activates the granule cell. Taken together, the data from paired intracellular recording and studies of neuromodulators suggests that granule cells will be activated best when there is septohippocampal tone or brainstem activation. That would mean times of spatial exploration and/or flight/fright such as when a predator nears. Indeed, mossy cells do discharge during theta rhythm (Soltesz et al., 1993) and it was predicted that they would have an important role based on these data and others *in vivo* data (Buckmaster and Schwartzkroin, 1994; Bragin et al., 1995; Penttonen et al., 1997). These studies are consistent with the idea that the mossy cell is important to relay to granule cells information about the environment or context.

## Conclusion

Mossy cells of the DG are glutamatergic neurons that have intrinsic and circuit properties that make them ideal to activate granule cells, which is likely to be necessary because the granule cells are quiescent, hyperpolarized neurons. That quiescence seems necessary for cognitive functions such as pattern separation but leaves granule cells at risk of suboptimal activation. Mossy cells could serve to inhibit local granule cells so they are not activated too much by an input, supporting pattern separation, but activate granule cells in distal DG lamellae to relay or “broadcast” information that might otherwise be undetected, which may support heterassociative function. For this potentially important role, mossy cells may pay a “price”—vulnerability to insults or injuries that are associated with release of high concentrations of glutamate from the mossy fibers.

### Conflict of interest statement

The authors declare that the research was conducted in the absence of any commercial or financial relationships that could be construed as a potential conflict of interest.
